# Phosphorylation at Ser473 regulates heterochromatin protein 1 binding and corepressor function of TIF1beta/KAP1

**DOI:** 10.1186/1471-2199-9-61

**Published:** 2008-07-01

**Authors:** Chiung-Wen Chang, Han-Yi Chou, Yu-Sheng Lin, Kuo-Hsiang Huang, Ching-Jin Chang, Tsui-Chun Hsu, Sheng-Chung Lee

**Affiliations:** 1 Institute of Molecular Medicine, College of Medicine, National Taiwan University, Taipei, Taiwan; 2 Institute of Biological Chemistry, Academia Sinica, Taipei, Taiwan; 3 Institute of Clinical Medicine, College of Medicine, National Taiwan University, Taipei, Taiwan

## Abstract

**Background:**

As an epigenetic regulator, the transcriptional intermediary factor 1β (TIF1β)/KAP1/TRIM28) has been linked to gene expression and chromatin remodeling at specific loci by association with members of the heterochromatin protein 1 (HP1) family and various other chromatin factors. The interaction between TIF1β and HP1 is crucial for heterochromatin formation and maintenance. The HP1-box, PXVXL, of TIF1β is responsible for its interaction with HP1. However, the underlying mechanism of how the interaction is regulated remains poorly understood.

**Results:**

This work demonstrates that TIF1β is phosphorylated on Ser473, the alteration of which is dynamically associated with cell cycle progression and functionally linked to transcriptional regulation. Phosphorylation of TIF1β/Ser473 coincides with the induction of cell cycle gene *cyclin A2 *at the S-phase. Interestingly, chromatin immunoprecipitation demonstrated that the promoter of *cyclin A2 *gene is occupied by TIF1β and that such occupancy is inversely correlated with Ser473 phosphorylation. Additionally, when HP1β was co-expressed with TIF1β/S473A, but not TIF1β/S473E, the colocalization of TIF1β/S473A and HP1β to the promoters of *Cdc2 *and *Cdc25A *was enhanced. Non-phosphorylated TIF1β/Ser473 allowed greater TIF1β association with the regulatory regions and the consequent repression of these genes. Consistent with possible inhibition of TIF1β's corepressor function, the phosphorylation of the Ser473 residue, which is located near the HP1-interacting PXVXL motif, compromised the formation of TIF1β-HP1 complex. Finally, we found that the phosphorylation of TIF1β/Ser473 is mediated by the PKCδ pathway and is closely linked to cell proliferation.

**Conclusion:**

The modulation of HP1β-TIF1β interaction through the phosphorylation/de-phosphorylation of TIF1β/Ser473 may constitute a molecular switch that regulates the expression of particular genes. Higher levels of phosphorylated TIF1β/Ser473 may be associated with the expression of key regulatory genes for cell cycle progression and the proliferation of cells.

## Background

Transcriptional intermediary factor TIF1β and heterochromatin protein 1 profoundly impact the regulation of the structure and function of chromatin [1]. The heterochromatin protein 1 (HP1) family of proteins (HP1α, HP1β, and HP1γ) participates in gene silencing by forming heterochromatic structures [2,3]. HP1 exhibits distinct nuclear localization patterns: HP1α nassociates with centromeres while HP1β and HP1γ are largely localized in distinct nuclear regions. The nuclear arrangement of HP1 proteins and TIF1β is differentiation pathway-specific, and appears to be more important than changes in the levels of these proteins, which are relatively stable during all of the induced differentiation processes [4,5].

HP1 proteins comprise an N-terminal chromodomain, a C-terminal chromoshadow domain and a hinge domain [3,6,7]. The chromodomain functions as a protein interaction domain, bringing together different proteins in multi-protein complexes and recruiting them to heterochromatin. This domain binds to particular proteins which contain an HP1 box (PXVXL) and function at the transcriptional level [2,8-10]. The chromoshadow domain mediates the formation of homodimer. TIF1β interacts with the chromodomain of dimeric HP1β via the HP1 box of TIF1β in the HP1-interaction domain [6]. The TIF1β ntranscriptional repression activity depends on the interaction between TIF1β and HP1 [11]. This interaction is essential in the relocation of TIF1β from euchromatin to heterochromatin that accompanies the differentiation of primitive endoderm-like cells [12]. TIF1β is proposed to function as a universal co-repressor protein for the KRAB zinc finger protein (KRAB-zfp) superfamily of transcriptional repressors. The recruitment of HP1 proteins by the KRAB-TIF1β complex to specific loci within the genome via the formation of heterochromatin-like complexes may thus silence gene activity. Gene-specific repression may be a consequence of the formation of such complexes [13].

It has been reported that TIF1β directly interacts with the histone methyltransferase SETDB1, which methylates specifically histone H3/Lys9 within euchromatin [14]. Depletion of endogenous levels of TIF1β by siRNA significantly inhibited the KRAB-mediated transcriptional repression of a chromatin template and inhibited cell cycle progression. Cell death may occur if TIF1β is severely depleted by siRNA knockdown [unpublished observations, [15-17]]. Similarly, the knock-down of cellular levels of HP1 proteins and SETDB1 by siRNA attenuated KRAB-TIF1β repression. The physiological targets and functions of TIF1β remain unclear. Its interactions with chromatin modification factors such as HDAC1, SETDB1 and HP1 correlate with its activities in regulating the chromatin structure and heterochromatin formation, resulting in epigenetic silencing of reporter genes [18]. A TIF1β-containing multiprotein complex regulates various protein activities or genes [19]. The activity of TIF1β may be regulated by posttranslational modifications. For example, TIF1β is rapidly phosphorylated by members of the phosphatidylinositol-3 kinase-like family of kinases following DNA damage. Phosphorylated TIF1β colocalizes with numerous damage response factors at DNA lesions [20]. Recent genetic and proteomic studies in mice have shown that TIF1β is a developmental regulatory protein performing cellular function(s) that are critical to early embryonic development, and is a component of the interactive protein network for the pluripotency of embryonic stem cells [21,22]. The binding of the TIF1β corepressor to the retrovirus primer binding site is responsible for the epigenetic silencing of retrovirus transcription [17]. Therefore, the interaction between TIF1β and HP1 or other transcription factors may account for the regulation of gene expression.

Phosphorylation of both human and mouse TIF1β/Ser473 has been identified by nuclear phosphoprotein analysis of HeLa and WEHI-231 cells [23,24]. Ser473 is located in the HP1-interacting domain of TIF1β – close to the HP1 box (amino acids 486–490, PXVXL). The conservation of TIF1β/Ser473 phosphorylation in various cell lines from different species motivated this investigation of the functional significance of this modification.

The coil-coiled domain of TIF1β binds to E2F1 and inhibits its activity [16]. The induction of *cyclin A*, *Cdc2 *and *Cdc25A *genes depends on E2F [25,26]. Cyclin A is a cell cycle-regulating protein that participates in S-phase control and mitosis in mammalian somatic cells. The promoter of *cyclin A *is repressed during the G1 phase of the cell cycle and is activated at S-phase entry [27]. Cdc2 permits the transition from G1 through S in conjunction with cyclin E [28]. These E2F downstream genes are important to normal cell cycle progression [29].

This report shows that TIF1β participates in the regulation of *cyclin A2*, *Cdc2 *and *Cdc25A *gene expression. This regulation depends on the interaction between TIF1β and HP1β, which is itself regulated by the phosphorylation state of TIF1β/Ser473. The experimental results suggest that the TIF1β/Ser473-unphosphorylated form binds more strongly than the phosphorylated form to the promoters of *Cyclin A2*, *Cdc2*, and *Cdc25A *genes. The phosphorylation/de-phosphorylation of TIF1β/Ser473 may serve as a molecular switch regulating its interaction with HP1β and gene expression.

## Results

### Characterization of TIF1β and phosphorylated TIF1β/Ser473 antibodies

**Figure 1:**
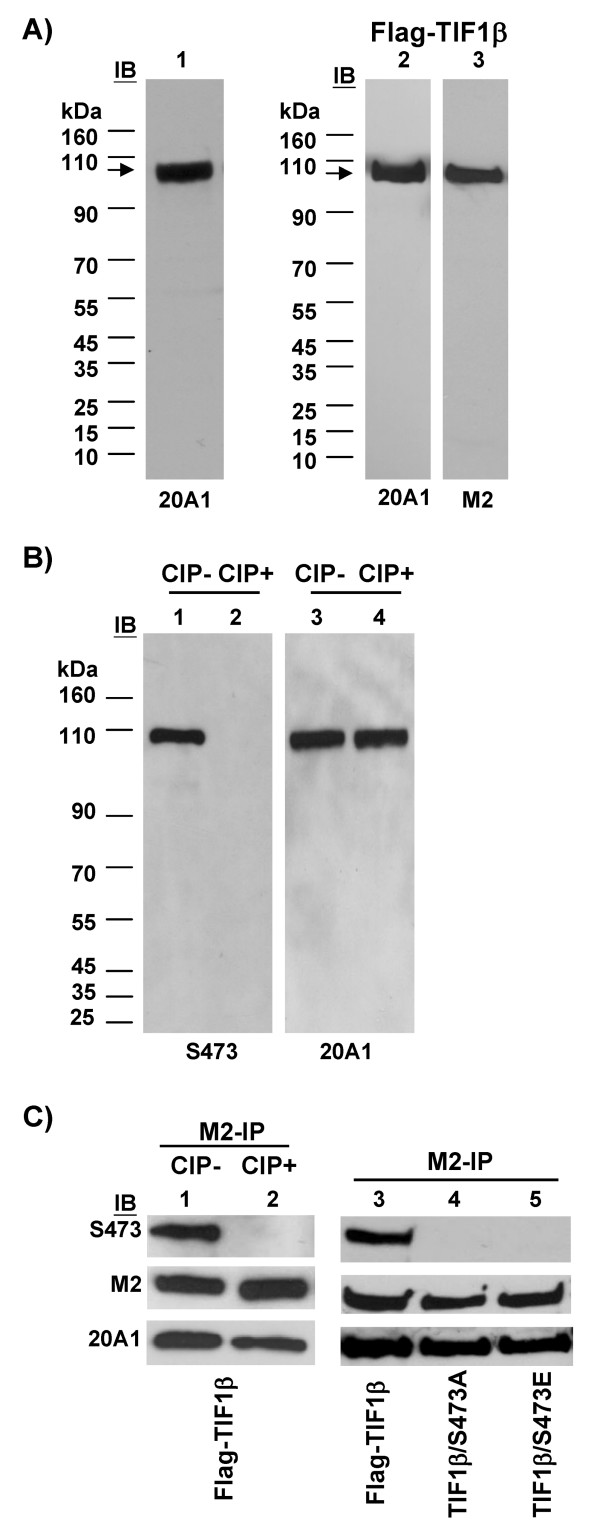
**Characterization of monoclonal anti-TIF1β (20A1) and rabbit anti-phosphorylated TIF1β/Ser473 (S473) antibodies**. (**A**) HeLa cell lysate were probed with anti-TIF1β monoclonal antibody, 20A1 (lane 1). Lysate from 293T cells transfected with FLAG-TIF1β were probed with 20A1 antibody (lane 2) or anti-Flag monoclonal antibody, M2 (lane 3). (**B**) Whole cell extracts were prepared from 293T cells and incubated in the absence (lanes 1 and 3) or presence (lanes 2 and 4) of CIP (30 U) for 30 min at 37°C. Western blotting was performed with antibodies indicated. (**C**) M2-beads purified FLAG-TIF1β from 293T cells were treated without (lane 1) or with (lane 2) CIP (30U) for 30 min. Western blot was probed with rabbit anti-TIF1β/Ser473 antibody (S473), anti-Flag antibody (M2), and anti-TIF1β antibody (20A1). Wild-type FLAG-TIF1β (lane 3), FLAG-TIF1β/S473A (lane 4) or FLAG-TIF1β/S473E (lane 5), were immunoprecipitated by M2-beads, resolved in SDS-PAGE, transferred onto membrane and probed with antibodies as indicated.

Western blotting of interphase HeLa cell extract was performed to characterize the specificity of monoclonal antibody to TIF1β, clone 20A1. A specific band of 100 kDa was detected (Figure 1A). 293T cell-expressed FLAG-TIF1β was recognized either by 20A1 or monoclonal anti-FLAG antibody, M2 (Figure 1A), demonstrating that monoclonal antibody 20A1 is specific to TIF1β.

The specificity of rabbit anti-phospho-Ser473 antibody was characterized by Western blotting of interphase 293T cell extract with rabbit anti-TIF1β/phospho-Ser473 antibody (S473) or 20A1 monoclonal antibody. The phosphorylated TIF1β/Ser473 signal was diminished after calf intestine alkaline phosphatase (CIP) treatment (Figure 1B). Ectopically-expressed FLAG-TIF1β was immunoprecipitated from 293T cells with M2 beads and treated with CIP to examine whether the signal was due to the phosphorylation of TIF1β/S473. 20A1 and S473 antibodies both recognized FLAG-TIF1β, but the signal recognized by the S473 antibody disappeared upon CIP treatment (Figure 1C). Unlike the FLAG-TIF1β, mutants FLAG-TIF1β/S473A and FLAG-TIF1β/S473E were not detected by S473 antibody (Figure 1C). These data demonstrate that while 20A1 or S473 antibodies recognize the same protein, TIF1β, the S473 antibody specifically recognizes phosphorylated TIF1β/S473.

### Phosphorylation of TIF1β/Ser473 is dynamically regulated during cell cycle progression

**Figure 2:**
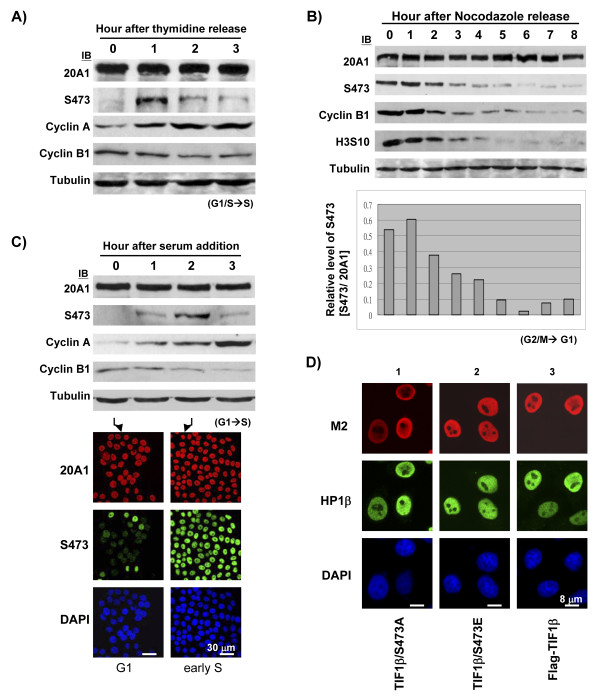
**Phosphorylation of TIF1β/Ser473 is dynamically regulated during cell cycle progression**. (**A**) HeLa cells were arrested at G1/S boundary by double thymidine block and released into S phase for three hours. The levels of TIF1β and phosphorylated TIF1β/Ser473 were probed with 20A1 and S473 antibodies. The protein levels of cyclin A, cyclin B1, or α-tubulin were also shown. (**B**) HeLa cells were synchronized by nocodazole (1 μM) treatment for 16 hours. Mitotic shake off cells were collected and released for 8 hours. The levels of TIF1β and phosphorylated TIF1β/Ser473 were probed with 20A1 and S473 antibodies. The protein levels of cyclin B1, phosphorylated Histone H3 S10 (H3S10), or α-tubulin were also probed as indicated. The relative phosphorylation level of TIF1β/Ser473 was compared by quantitative determination of the images from Western blots (by ImageGauge software, normalized to individual TIF1β level) at each time point (shown in the lower panel). (**C**) HeLa cells were arrested at G1 phase by serum starvation for three days and released to S phase by serum addition. The levels of TIF1β and phosphorylated TIF1β/Ser473 were probed with 20A1 and S473 antibodies. The protein levels of cyclin A, cyclin B1, and α-tubulin were also probed as indicated. HeLa cells at 0 hr (G1) or 2 hours after serum addition (early-S) were immunostained with 20A1 (red) and S473 (green) antibodies, DNA was counter stained with DAPI (blue). (**D**) Immunostaining of ectopically expressed FLAG-TIF1βs. FLAG-TIF1β/S473A (column 1) or FLAG-TIF1β/S473E (column 2) and wild-type FLAG-TIF1β (column 3) were transiently transfected into HeLa cells. Cells were fixed at 36 hours after transfection and co-stained with M2 (red) and HP1β (green) antibodies. DNA was counter stained with DAPI (blue). Control immunostaining was negative when performed with secondary antibody alone (data not shown). Bars in C represent 30 μm and bars in D show 8 μm.

Although TIF1β is known to be a general corepressor protein, few studies have suggested that TIF1β has a critical function in cell cycle progression. The phosphorylation of TIF1β/Ser473 was identified in HeLa and WEHI cells at interphase [23,24]. Since Ser473 is located near the HP1-binding motif, an intriguing question arises: what is the functional consequence, if any, of the phosphorylation of TIF1β/Ser473? To elucidate the phosphorylation state of TIF1β/Ser473 during cell cycle progression, Western blotting was performed with lysates from synchronized HeLa cells. Cells were synchronized to G1/S transition using double thymidine block. Phosphorylated TIF1β/Ser473 was almost undetectable at the G1/S boundary under the thymidine block (Figure 2A, 0 hr, S473) but the phosphorylation of TIF1β/Ser473 was maximal 1 hour post-release from the thymidine block, during early S phase (Figure 2A, 1 hr, S473). The level of phosphorylated TIF1β/Ser473 decreased thereafter for 2 hours during the mid to late S-phase cell cycle progression. The level of total TIF1β remained relatively constant (Figure 2A, 20A1). The elevated cyclin A and decreased cyclin B evidenced the G1/S to S cell cycle progression of thymidine release (Figure 2A).

HeLa cells were synchronized to prometaphase by nocodazole treatment. The level of phosphorylated TIF1β/Ser473 was determined from HeLa extracts collected from 0 to 8 hours after nocodazole were removed. The phosphorylated TIF1β/Ser473 level was highest during mitosis (Figure 2B, 0–2 hours, S473) and decreased thereafter through G1 phase (Figure 2B). The lower panel of Figure 2B shows the level of phosphorylated TIF1β/Ser473 in relation to that of total TIF1β at each time point. The M-phase marker cyclin B or phosphorylated histone H3S10 identify specific stages of mitotic release (Figure 2B).

To further evaluate the phosphorylation state of TIF1β/S473 from G1 to S phase, HeLa cells were serum starved for three days to arrest in G1 phase. The level of phosphorylated TIF1β/Ser473 was almost non-detectable in G1 phase (Figure 2C, 0 hr, S473), reached a maximum 2 hours after serum was added and declined thereafter (Figure 2C, 2 hr, S473). The gradual increase in the level of cyclin A or decrease in the level of cyclin B demonstrated the specific cell cycle stage from G1 to S (Figure 2C). This dynamic, biphasic (S and M phases) appearance of phosphorylated TIF1β/Ser473 suggests that TIF1β may be involved in regulating the cell cycle by the switching on and off of Ser473 phosphorylation.

Various cell cycle regulated proteins may exhibit changed activity when phosphorylation modulates their stability. The total protein level of TIF1β remained approximately constant while the level of phosphorylated TIF1β/Ser473 fluctuated throughout the cell cycle. To further demonstrate this dynamic fluctuation of phosphorylated TIF1β/Ser473 level, HeLa cells were immunostained using S473 antibody. The level of phosphorylated TIF1β/Ser473 in the cells 2 hr after serum addition markedly exceeded that in the serum-starved cells (Figure 2C, immunofluorescence staining, compare early S and G1, S473). The protein level and distribution of TIF1β during G1 were the same as those of early S phase (Figure 2C, immunofluorescence staining, compare 20A1). Furthermore, in later experiments using over-expressed FLAG-TIF1βs, similar nuclear distribution patterns of over-expressed wild-type FLAG-TIF1β, FLAG-TIF1β/S473A and FLAG-TIF1β/S473E were observed (Figure 2D, compare M2). HP1β staining also showed nuclear localization with over-expressed FLAG-TIF1βs (Figure 2D, compare HP1β in transfected cells).

As a well known corepressor, TIF1β regulates gene expression through direct binding to HP1 proteins in numerous transcriptional-repressed loci. Thus, the immunostaining data further indicate that TIF1β might control its co-regulator function in the nuclear microenvironment via Ser473 phosphorylation/dephosphorylation.

### Phosphorylation of TIF1β/Ser473 compromises interaction between HP1β and TIF1β

**Figure 3:**
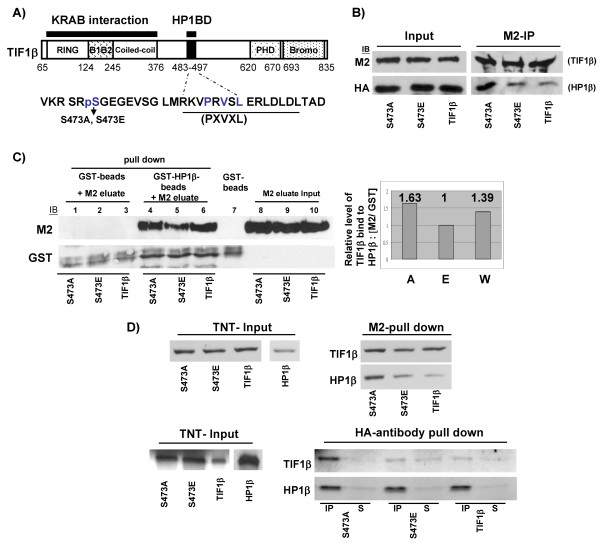
**Interaction between TIF1β and HP1β is compromised by phosphorylation of TIF1β/Ser473**. (**A**) Schematic representation of domain organization of TIF1β protein. The position of Ser473 relative to the PXVXL motif is shown. (**B**) Western blot results of co-immunoprecipitation of over-expressed FLAG-TIF1β and HA-HP1β. FLAG-TIF1β/S473A, FLAG-TIF1β/S473E and wild-type FLAG-TIF1β were co-expressed with HA-HP1β into 293T cells. The level of ectopically expressed FLAG-TIF1βs (Input, M2) or M2-beads immunoprecipitated FLAG-TIF1βs (M2-IP, M2) was probed with M2 antibodies (TIF1β, M2). The level of ectopically expressed HA-HP1β (Input, HA) or M2-beads co-immunoprecipitated HA-HP1βs (M2-IP, HA) was probed with monoclonal anti-HA antibodies (HP1β, HA). (**C**) *In vitro *GST pull-down assay of TIF1βs by recombinant GST-HP1β. Wild-type and mutant FLAG-TIF1βs were expressed in 293T cells. These FLAG-TIF1βs were immunoprecipitated by M2 beads and eluted with M2 peptide. The levels of FLAG-TIF1βs from 1/10 input for each pull down assay were shown (lanes 8–10). Eluted FLAG-TIF1βs were incubated with GST-beads alone (lanes 1–3) or incubated with recombinant GST-HP1β bound glutathione-beads (lanes 4–6). The levels of GST-HP1β and FLAG-TIF1βs in each pull down assay were shown (lanes 1–6). Quantitation of the Western blots (by ImageGauge software) from each pull down sample (lanes 4–6) showing the relative level of FLAG-TIF1βs (A, TIF1β/S473A; E, TIF1β/S473E; W, wild-type TIF1β) pulled down by GST-HP1β, after normalization to individual GST-HP1β levels (right panel). (**D**) Interaction between *in vitro *translated FLAG-TIF1βs and HA-HP1β. Upper panel, *In vitro *translated [^35^S] FLAG-TIF1βs and [^35^S]-HA-HP1β were incubated for 30 min before being immunoprecipitated by M2 beads in the presence of 0.5 M NaCl. The immunoprecipitates were resolved by SDS-PAGE and followed by autoradiography to visualize HA-HP1β (upper right panel, M2-pull down). Lower panel, *In vitro *translated [^35^S] FLAG-TIF1βs and [^35^S] HA-HP1β were pulled down by protein-G coupled anti-HA antibody. The immunoprecipitated FLAG-TIF1βs are shown in the lower right panel (TIF1βs, IP). Supernatants after immunoprecipitation are also shown, as indicated (S).

The Ser473 is located close to the PXVXL (aa 486–490)-containing HP1 box (aa 483–497) (Figure 3A). To elucidate the role of TIF1β in regulating gene expression, we examined whether its interaction with HP1β was affected by the phosphorylation of S473. Immunoprecipitation and Western blotting experiments were performed using 293T cells that were co-transfected with FLAG-TIF1β (TIF1β), FLAG-TIF1β/S473A (S473A) or FLAG-TIF1β/S473E (S473E) and HA-HP1β. The interaction between FLAG-TIF1β/S473A and HP1β was stronger than that between FLAG-TIF1β/S473E or FLAG-TIF1β and HP1β (Figure 3B). To demonstrate that the reduced phosphorylation level of TIF1β/Ser473 was related to its stronger interaction with HP1β, a pull-down assay was performed using purified GST-HP1β from *E. coli *and FLAG-TIF1β, FLAG-TIF1β/S473A or FLAG-TIF1β/S473E from 293T cells. GST-HP1β interacted with non-phosphorylated-mimetic FLAG-TIF1β/S473A 1.6 times more effectively than with phosphorylated-mimetic FLAG-TIF1β/S473E (Figure 3C). The similarity of the level of interaction between wild-type FLAG-TIF1β and GST-HP1β and that of FLAG-TIF1β/S473A and GST-HP1β (1.39 vs. 1.63) may reflect a lower phosphorylation level of wild-type FLAG-TIF1β here. To verify that TIF1β/Ser473 phosphorylation compromises the interaction between TIF1β and HP1β, M2 or HA antibody pull-down assays were conducted using *in vitro *translated HA-HP1β and FLAG-TIF1β, FLAG-TIF1β/S473A, or FLAG-TIF1β/S473E. The results of the M2 pull-down and the HA antibody pull-down assay indeed demonstrated that the interaction between HA-HP1β and FLAG-TIF1β/S473A is much stronger than that between FLAG-TIF1β/S473E and FLAG-TIF1β (Figure 3D). Taken together, these data demonstrate that the phosphorylation state of TIF1β/Ser473 influences the interaction between TIF1β and HP1β both *in vivo *and *in vitro*. When TIF1β/Ser473 is phosphorylated, its interaction with HP1β is compromised.

Although the TIF1β co-repressor function is known to be related to HP1β, few studies have addressed the specific gene targets of TIF1β. TIF1β/S473 phosphorylation is up-regulated during the S phase in HeLa cells and the interaction between HP1β and TIF1β is compromised when Ser473 is phosphorylated. These observations suggest that the phosphorylation of TIF1β/Ser473 may regulate gene expression by abolishing its interaction with HP1β.

### TIF1β regulates key genes expression during G1 to S-phase cell cycle progression

**Figure 4:**
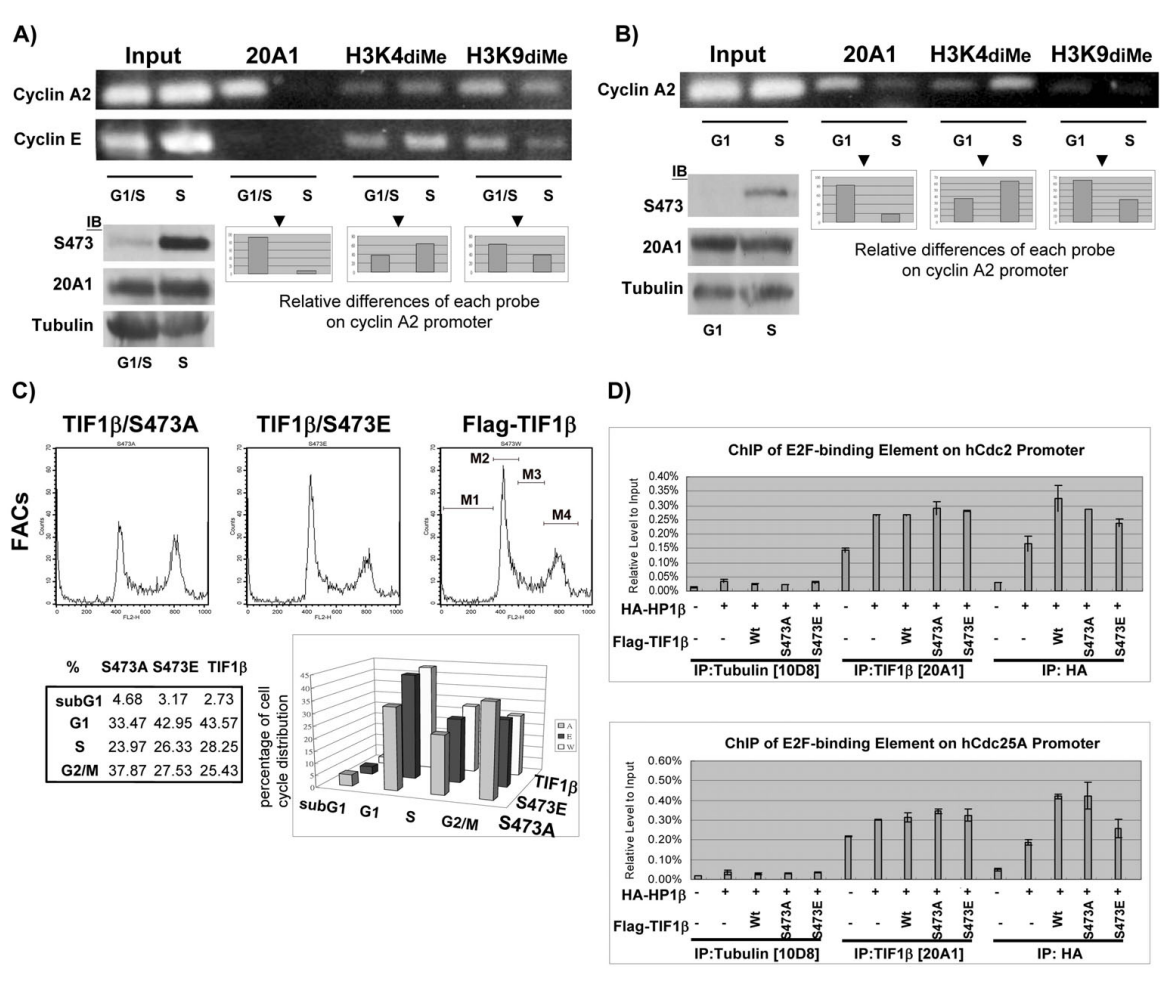
**TIF1β is involved in the regulation of expression of E2F downstream genes, *cyclin A2, Cdc2 *and *Cdc25A*.** (A) Binding of TIF1β to the promoter of *cyclin A2 *gene is correlated with its repression. HeLa cells were synchronized to G1/S boundary by double thymidine block and subsequently released for 1 hour to the early S phase. ChIP was performed as described in the Materials and Methods section. Equal loadings of input derived from G1/S and S cells were used for ChIP experiments (upper panels), using 20A1, H3K4diMe, and H3K9diMe antibodies. PCR was performed with input samples (1/50) and ChIP eluates. Relative differences between G1/S and S phase of each indicated probe on *cyclin A2 *promoter are shown in the lower right panels (comparing the *cyclin A2 *signal of G1/S and S phase from each probe with the same total area, by ImageGauge software). The protein level of TIF1β (20A1), α-tubulin and phosphorylated TIF1β/Ser473 (S473) from formaldehyde cross-linked samples are shown in the lower left panel. PCR for *cyclin E *promoter was used as a control (upper panel). (**B**) HeLa cells were arrested at G1 phase by serum starvation and released into early S phase by addition of serum to the culture for 2 hours. ChIP was performed as in (A) with 20A1, anti-H3K4diMe and anti-H3K9diMe antibodies (upper panel). Relative differences between G1 and S phase of each indicated probe on *cyclin A2 *promoter are shown in the lower right panels (by ImageGauge, as in A), while the protein levels are shown in the lower left panel. (**C**) Flow cytometry analysis. FLAG-TIF1β/S473A, FLAG-TIF1β/S473E and wild-type FLAG-TIF1β were over-expressed in 293T cells, collected 36 hours post-transfection and analyzed by flow cytometry after staining with propidium iodide, and the FCM histograms are shown in the upper panels. Cell cycle phase distributions (%) of TIF1β over-expressing cells were analyzed with the CellQuest software and shown in the lower left panel, according to the gated region from the upper panel (M1, subG1; M2, G1; M3, S; and M4, G2/M, by CellQuest program), and the bar charts represented the cell cycle distribution (in total for 100%) are shown in the lower right panel (by Microsoft Excel). (**D**) The HP1β complex is preferentially associated with the promoters of *Cdc2 *and *Cdc25A *in TIF1β/S473A over-expressing interphase cells of HEK293T. FLAG-TIF1β, FLAG-TIF1β/S473A, FLAG-TIF1β/S473E and HA-HP-1β were ectopically expressed in HEK 293T cells for 36 hours and subjected to ChIP with the indicated antibodies (anti-tubulin, 10D8; anti-TIF1β, 20A1; anti-HA, HA). DNA from the immunoprecipitated chromatin was quantified by real-time PCR. Each result was normalized to input and the relative level to the transfected vector control. Results for the *Cdc2 *promoter (upper panels) and *Cdc25A *promoter (lower panels) are shown.

Chromatin immunoprecipitation (ChIP) experiments were used to study the possible involvement of Ser473-phosphorylated/dephosphorylated TIF1β in the expression of HeLa cell cycle-specific genes during the transition from G1 phase to S phase. TIF1β/phospho-Ser473 was almost undetectable in the G1 phase, and climbed thereafter to reach maximum level in early S phase. As *Cyclin A2 *is known to be expressed in early S phase but not in G1 phase, a reasonable question arises: Is the TIF1β/Ser473 phosphorylation correlated with *cyclin A2*'s expression? PCR primers encompassing the E2F site were used to detect DNA fragments corresponding to the promoter region of *cyclin A2*. A much higher level of TIF1β was associated with the *cyclin A2 *promoter at the G1/S boundary than in early S phase (Figure 4A, upper panel). These results are closely correlated with the rise in phosphorylated TIF1β/Ser473 in the early S phase, but not at the G1/S boundary (Figure 4A, lower left panel). Histone H3 K9diMe was associated with the silencing of gene expression. The ChIP results demonstrated that the level of H3K9diMe correlated strongly with that of TIF1β. However, histone H3K4diMe is known to associate with actively transcribed genes. Its association with the promoter region of *cyclin A2 *was inversely correlated with that of TIF1β. Quantitative ChIP results comparing the *cyclin A2 *level in G1/S and S phase are presented under the panel of PCR results obtained using each probe. As a control, TIF1β was not associated with the promoter of *cyclin E *(Figure 4A, upper panel). TIF1β/phospho-Ser473 was also elevated 2 hours after serum was added (Figure 2C). ChIP was thus used to measure the level of TIF1β bound to the *cyclin A2 *promoter in G1-phase HeLa cells that had been starved of serum for three days or S-phase HeLa cells that had been released from serum starvation. More TIF1β in the G1 phase cells was associated with the promoter region of *cyclin A2 *(Figure 4B, upper panel). This finding is closely correlated with the elevated level of phosphorylated TIF1β/Ser473 in the S phase but not in the G1 phase (Figure 4B, lower left panel). Quantitative ChIP results, comparing *cyclin A2 *levels in the G1 and S phase, are presented beneath the PCR results obtained using each probe. Taken together, these results indicate that the association of TIF1β with the promoter of *cyclin A2 *is correlated with silencing while dissociation is correlated with derepression of the promoter.

Immunostaining showed that over-expression of FLAG-TIF1β, FLAG-TIF1β/S473A, and FLAG-TIF1β/S473E did not alter their nuclear localization (Figure 2D). A flow cytometric analysis was performed to test further the effects of over-expressed FLAG-TIF1β, FLAG-TIF1β/S473A and FLAG-TIF1β/S473E on cell cycle progression. Over-expression of the FLAG-TIF1β/S473A mutant caused an accumulation of cells stalled at G2/M in 293T cells (Figure 4C), while the profiles of cell cycle progression were similar in 293T cells over-expressing FLAG-TIF1β and FLAG-TIF1β/S437E, suggesting that the phosphorylation state of TIF1β/Ser473 affects cell cycle progression. Quantitative real-time PCR analysis of 293T cells revealed half the amount of cyclin A2 mRNA in the FLAG-TIF1β/S473A-overexpressing cells compared to the FALG-TIF1β or FALG-TIF1β/S473E-expressing cells (data not shown). The phenotype of the G2/M stalls and the reduction of the cyclin A2 mRNA level in TIF1β/S473A over-expressing 293T cells are consistent with observations made in a GFP-cyclin A2 siRNA knockdown experiment published by Kenrick et al. [29]. Taken together, these results suggest that disruption of TIF1β/Ser473 phosphorylation may influence cell cycle-regulated gene expression, and that *cyclin A2 *may be one of the TIF1β indirect target genes. These data also demonstrate that the Ser473 phosphorylation/dephosphorylation status of TIF1β may regulate cell cycle progression.

To further examine the association of unphosphorylated TIF1β/Ser473 with other cell cycle-regulated genes, such as *Cdc2 *and *Cdc25A*, quantitative ChIP experiments were conducted with HEK293T cells that had been transfected with HA-HP1β and FLAG-TIF1β, FLAG-TIF1β/Ser473A, and FLAG-TIF1β/Ser473E. When ChIP was performed with HA monoclonal antibody, the association of HP1β with the promoters of *Cdc2 *or *Cdc25A *in FLAG-TIF1β/Ser473A over-expressing cells was stronger than in FLAG-TIF1β/Ser473E-over-expressing cells (Figure 4D). When ChIP was performed using 20A1 (which recognizes the N-terminal of TIF1β), no obvious difference between FLAG-TIF1β/Ser473A and FLAG-TIF1β/Ser473E was observed. Collectively, these results reveal that over-expressed HP1β and TIF1β/Ser473A may form a stronger complex and preferentially associate with the promoter regions of *Cdc2 *and *Cdc25A *genes more than over-expressed HP1β and TIF1β/Ser473E in interphase HEK293T cells.

### Phosphorylation of TIF1β Ser473 in S phase is mediated by PKC pathway

**Figure 5:**
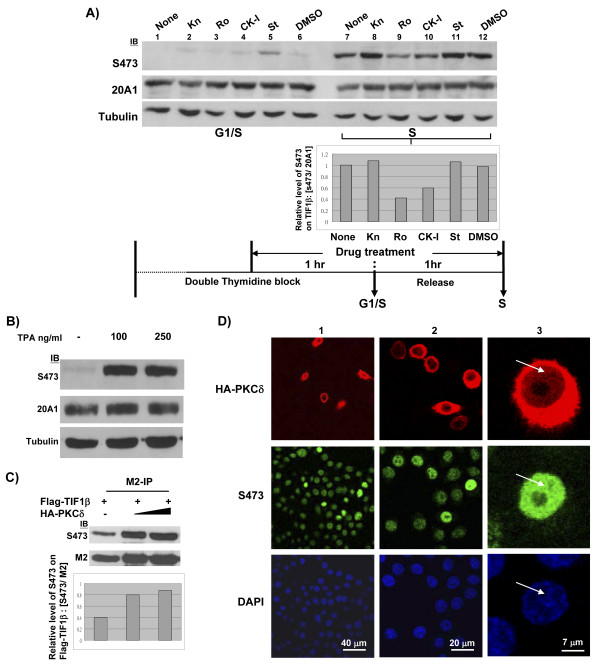
**PKC is involved in the phosphorylation of TIF1β/Ser473 in early S phase**. (**A**) HeLa cells were synchronized with double thymidine block at G1/S phase and treated with various kinase inhibitors before being released for 1 hour to early S-phase (experimental procedures are depicted in the bottom scheme). Lanes 1–6, Western blot of whole cell extracts of cells arrested at G1/S boundary. Lanes 7–12, Western blot of early S-phase extracts. The inhibitors used were CaMK-II inhibitor KN93 (KN, 1 μM), PKC inhibitor Ro-31-8220 (Ro, 100 nM), CK1 inhibitor D4476 (CK-I, 10 μM,), staurosporin (St, 20 nM), and DMSO as a control. The levels of TIF1β (20A1), phosphorylated TIF1β/Ser473 (S473) and α-tubulin are shown. Quantitative results of the Western blots in early S phase (by ImageGauge software) showing the relative TIF1β/Ser473 phosphorylation level in various kinase inhibitor treatment, after normalization to individual TIF1β level. (**B**) Phosphorylated TIF1β/Ser473 was highly elevated in TPA-treated HeLa cells. Interphase HeLa cells were treated with 100 ng/ml or 250 ng/ml TPA for 1 hour, and whole cell extracts were prepared for Western blot. (**C**) Phosphorylation of TIF1β/Ser473 in 293T cells was highly induced by over-expressed HA-PKCδ. 293T cells were cotransfected with 1 μg of FLAG-TIF1β plasmid and HA-PKCδ plasmids (1 or 2 μg). FLAG-TIF1β was immunoprecipitated by M2-beads 36 hours post-transfection and probed with antibody to phosphorylated TIF1β/Ser473. Quantitative results (by ImageGauge software) show the relative TIF1β/S473 phosphorylation level in the presence of various amounts of transfected PKCδ plasmid, after normalization to individual TIF1β level. (**D**) Phosphorylation of TIF1β/Ser473 was specifically increased in HA-PKCδ transfected HeLa cells. HA-PKCδ transfected HeLa cells were fixed 36 hours post-transfection and co-stained with monoclonal anti-HA antibody (red) and TIF1β/S473 antibody (green). DNA was visualized by counter staining with DAPI (blue). Different maginification of images are shown in columns 1–3. The localization of HA-PKCδ or TIF1β in the nucleus is indicated (column 3, arrow). Bars in D represent: column 1, 40 μm; column 2, 20 μm; and column 3, 7 μm.

TIF1β/Ser473 phosphorylation is up-regulated in S phase. To identify which kinase(s) are involved in the phosphorylation of TIF1β/Ser473 in the S phase, we used Western blotting of cell extracts prepared from cells that had been treated with a panel of kinase inhibitors. Among the inhibitors tested, only the PKC inhibitor (Ro-31-8820) exhibited significant inhibition, while the CK1 inhibitor had a moderate effect on the phosphorylation of Ser473 (Figure 5A). An inhibitory peptide (a substrate-mimetic fragment from aa 466–478 of TIF1β) that contained TIF1β/S471A/S473A also blocked TIF1β/Ser473 phosphorylation (data not shown). Neither CaMK-II inhibitor nor staurosporine had any effect (Figure 5A). To confirm that the phosphorylation of TIF1β/Ser473 proceeds along the PKC pathway, the effect of 12-O-tetradecanoylphorbol-13-acetate (TPA) on TIF1β/Ser473 phosphorylation in interphase HeLa cells was tested. TPA treatment induced dramatic phosphorylation of TIF1β/Ser473 within 1 hour (Figure 5B). To further examine whether subtype-PKCδ is involved in the phosphorylation of TIF1β/Ser473, M2-immunoprecipitation and Western blotting with S473 antibody were performed using cell extracts that were prepared from FLAG-TIF1β and HA-PKCδ-cotransfected 293T cells. The phosphorylation level of TIF1β/Ser473 was double that of the control (Figure 5C). Immunostaining also revealed an increase of the TIF1β/Phospho-Ser473 signal in HA-PKCδ-transfected HeLa cells (Figure 5D). Although the majority of over-expressed PKCδ was located in the cytoplasm, a minor portion appeared in the nucleus (Figure 5D, column 3). Collectively, these results indicate that TIF1β/Ser473 phosphorylation in S phase could be mediated by the PKCδ pathway, and that TIF1β/Ser473 phosphorylation correlated with the expression of target genes by weakening TIF1β/HP1β interaction (and heterochromatin formation).

### Level of phosphorylated TIF1β/Ser473 is reduced in megakaryocytic differentiated K562 cells

**Figure 6:**
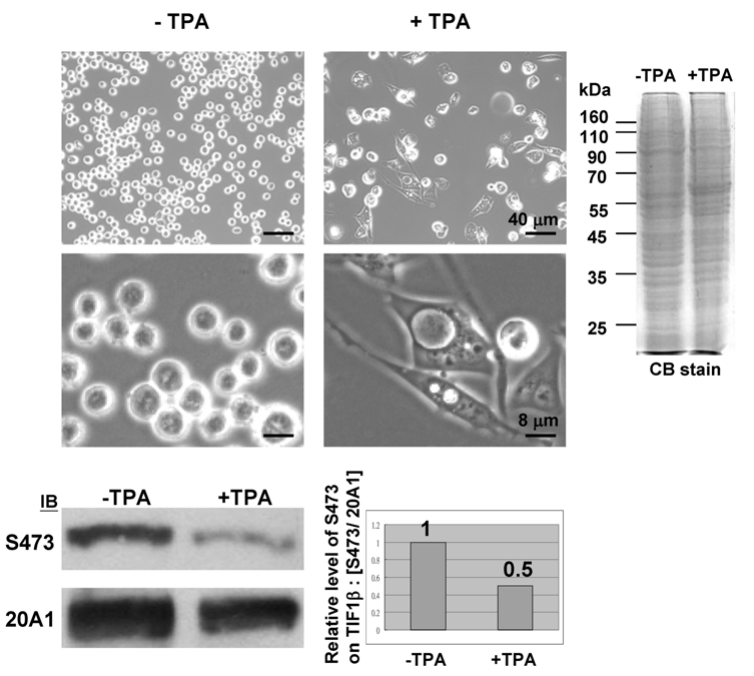
**The level of phosphorylated TIF1β/Ser473 is decreased in TPA-induced differentiated K562 cells**. K562 cells were treated with 10 ng/ml TPA for 4 days. Phase contrast images of control (left panels, TPA-) and differentiated (right panels, TPA+) K562 cells are shown. Equal amounts of proteins from these cells were collected (Coomassie Blue stain) and TIF1β (20A1) or phosphorylated TIF1β/S473 (S473) were detected by Western hybridization. The relative TIF1β/S473 level of proliferating or TPA-induced differentiated K562 cells (by ImageGauge software, after normalization to individual TIF1β level) is shown. Bars in phase contrast images represent 40 μm (upper panels) and 8 μm (lower panels).

TIF1β/Ser473 phosphorylation fluctuates during cell cycle progression. K562 cells were used as an inducible cell differentiation system to profile the level of phosphorylated TIF1β/Ser473 in both proliferating and differentiated cells. K562 is an erythroleukemic cell line that can undergo further differentiation in both megakaryocytic and erythroid lineages, depending on the stimulus [30,31]. It is a well-characterized system in which TPA stimulates the megakaryocytic development of K562. Western blotting was performed on extracts prepared from proliferating or TPA-induced differentiated K562 cells. The differentiated K562 cells exhibited megakaryocytic morphology (with lobulated nucleus, highly vacuolated, and histiocytoid), as demonstrated by phase contrast images (Figure 6). Although the total TIF1β protein level in differentiated K562 cells was lower than that in proliferating cells (Figure 6, CB stain and Western blot), the level of phosphorylated TIF1β/Ser473 was halved in megakaryocytic differentiated K562. Consistent with the observation that TIF1β promotes cell cycle progression by the up-regulation of its Ser473 phosphorylation, the amount of TIF1β/Ser473 phosphorylation may be utilized as a proliferation marker in cancer cells.

## Discussion

This investigation demonstrates that the phosphorylation of TIF1β/Ser473 by kinase(s) (such as PKCδ) may act as a molecular switch in the temporal regulation of gene expression. TIF1β/Ser473 is located close to the HP1 box, the PXVXL motif, which is responsible for the interaction between TIF1β and HP1, and its phosphorylation negatively affects this interaction. The dynamic phosphorylation of TIF1β/Ser473 suggests that this event may regulate the functions of TIF1β in cell cycle progression. The central question then is: How may the phosphorylation of TIF1β/Ser473 regulate TIF1β-mediated gene expression? We addressed this question by investigating the potential regulatory functions of TIF1β on key genes for cell cycle pregression and the effects of over-expressing phosphorylation-deficient or phosphomimetic mutants of TIF1β/Ser473 on cell cycle progression. TIF1β is proposed to function as a universal corepressor protein for the KRAB zinc finger protein (KRAB-zfp) superfamily of transcriptional repressors [32]. The recruitment of HP1 proteins by the KRAB-TIF1β complex to specific loci within the genome through the formation of heterochromatin-like complexes may silence gene activity.

### The phosphorylation of TIF1β/S473 compromises its interaction with HP1β in a manner that is related to cell cycle-regulated gene expression

The disruption of the interaction between HP1β and TIF1β by the phosphorylation of TIF1β/Ser473 (Figure 3) suggests that this phosphorylation/dephosphorylation of TIF1β/Ser473 may be the means of regulation of TIF1β- and HP1β-mediated gene silencing. The results of ChIP experiments suggest that the majority of TIF1β associated with the promoters of *cyclin A2 *in G1 phase cells is likely to be unphosphorylated TIF1β/Ser473. This conclusion is supported by two lines of evidence: (1) very low levels of phosphorylated TIF1β/Ser473 were observed in G1 cells, and (2) overexpressed FLAG-TIF1β/S473A bound to the promoters of *Cdc2 *and *Cdc25A *better than FLAG-TIF1β/S473E. When cells were released into the S phase, the association of unphosphorylated TIF1β/Ser473 with these promoters decreased, accompanying an increased level of phosphorylated TIF1β/Ser473. The dynamics of TIF1β/Ser473 phosphorylation and TIF1β-binding to the *cyclin A2 *promoter (during the G1 to S phase progression) indicate that un-phosphorylated TIF1β/Ser473 is responsible for silencing the *cyclin A2 *gene in the G1 phase (Figures 4A and 4B). The observation that HP1β interacted strongly with un-phosphorylated TIF1β/Ser473 is consistent with the ChIP results concerning the over-expressed recombinant variants, where over-expressed FLAG-TIF1β/S473A associated better with the promoter region of *Cdc2 *or *Cdc25A *than that did FLAG-TIF1β/S473E (Figure 4D).

The treatment of cells with cyclin A2 siRNA led to an accumulation of cells in prophase and mitosis to a degree similar to that observed for cyclin B1, consistent with the requirement of cyclin A for G1/S and G2/M transitions [29]. Interestingly, the ChIP results (Figures 4A and 4B) and the effects of over-expressed FLAG-TIF1β/Ser473A on cell cycle progression (accumulation of G2/M cells, Figure 4C) are consistent with published results for the siRNA knockdown of Cyclin A2 [29].

HP1β recruitment to E2F-binding element of *Cdc2 *and *Cdc25A *promoters was affected by the phosphorylation state of TIF1β/Ser473. The level of HP1β recruitment to *Cdc2 *or *Cdc25A *promoter was increased when wild-type FLAG-TIF1β or FLAG-TIF1β/S473A were ectopically expressed. This association was compromised by the phosphomimetic mutant, S473E, which suggests that HP1β recruitment is negatively regulated by phosphorylation of TIF1β/Ser473. Likewise, ectopically expressed HP1β resulted in elevated recruitment of wild-type FLAG-TIF1β or FLAG-TIF1β/S473A. These observations provide a clue that the recruitment of TIF1β and HP1β could work in a positive feedback manner.

The above results demonstrate that the dynamic interconversion between unphosphorylated and phosphorylated forms of TIF1β/Ser473 may be crucial in the regulation of gene expression during cell cycle progression. Despite extensive efforts to perform ChIP with rabbit anti-phosphorylated TIF1β/Ser473 antibody, no significant result was obtained. A likely explanation of this failure is that phosphorylated TIF1β/Ser473 is not associated with the promoter of *cyclin A2 *in the G1 phase or, alternatively, the epitope in the multiprotein complex is masked from antibody access.

The way in which TIF1β disassociates from the HP1-containing heterochromatin is unclear. However, the findings herein provide a molecular explanation, which involves PKC-mediated phosphorylation at TIF1β/Ser473 (Figure 5).

### Phosphorylated TIF1β/S473 level may serve as a proliferation marker

TIF1β transcriptional repression activity depends on the interaction between TIF1β and HP1β [11]. This interaction is essential to the relocation of TIF1β from euchromatin to heterochromatin that accompanies the differentiation of primitive endoderm-like cells [12]. TIF1β is known to interact differentially with HP1β and HP1γ in differentiated and non-differentiated cells [33]. In non-differentiated cells, TIF1β/HP1 interaction occurs only in euchromatin and selectively involves HP1β and HP1γ, but not HP1α. In differentiated cells, on the other hand, TIF1β selectively associates with HP1β in heterochromatin, while TIF1β and HP1γ interaction occurs only in euchromatin. These conclusions agree with the reduced level of phosphorylated TIF1β/Ser473 seen here in differentiated K562 cells (Figure 6). The results herein also revealed that un-phosphorylated TIF1β/Ser473 interacts more strongly with HP1β than its phosphorylated counterpart (Figure 4B). These results further suggest that the phosphorylation of TIF1β/Ser473 regulates the differential interaction between TIF1β and HP1β.

What may be the functional consequences of the phosphorylation of TIF1β/Ser473 and its consequent dissociation from HP1s must be addressed. TIF1β interacts with E2F [16], TRIP-Br and CBP/p300, and potentiates the co-activation of E2F-1/DP-1 by TRIP-Br protein [34]. Phosphorylated TIF1β/Ser473 may thus be involved in this tripartite functional interaction. This suggestion is supported by our findings that the induction of *cyclin A2 *is accompanied by an increased level of phosphorylated TIF1β/Ser473 and reduced binding of TIF1β to the promoter. A most provocative question that remains to be answered is whether the Ser473-phosphorylated TIF1β may interact preferentially with transcription factors and serve as a coactivator.

The results also demonstrated that the level of phosphorylated TIF1β/Ser473 peaks at early S- and M-phases. Two phosphorylation sites other than Ser473, Ser752 and Ser757, were also identified in the course of this investigation. Both sites are located in the bromodomain of TIF1β. The phosphorylation-deficient or phosphomimetic mutants of Ser757 did not influence the phosphorylation of Ser473 (Chang, unpublished results), suggesting that these phosphorylations may be independent events during mitosis. Other phosphorylation sites important for regulating the function of TIF1β have also been identified [35]. The functional consequences of Ser752 and Ser757 phosphorylation remain to be investigated.

### PKC mediated TIF1β/S473 phosphorylation may be involved in cell cycle progression

Numerous examples have demonstrated that PKCδ is centrally involved in cell proliferation and differentiation. The activation of PKCδ uniquely mediates insulin-induced proliferation: PKCδ is activated by insulin and interacts with insulin receptor and IRS [36-38]. Insulin-activated PKCδ interacts with 3-phosphoinositide-dependent protein kinase to regulate Protein Kinase B [39] and is responsible for STAT3 activation and keratinocyte proliferation [40]. Although the over-expressed PKCδ is mainly located in the cytoplasm (Figure 5D), it has been shown here (Figure 5D) and by Kajimoto et al. to partially localize to the nucleus [41]. The observation herein that the serum-stimulated phosphorylation of TIF1β/Ser473 correlates strongly with G1/S progression and *cyclin A2 *expression (Figure 4) uncovers a novel mechanism of PKCδ-mediated G1 to S phase cell cycle progression.

### Phosphorylation of TIF1β/Ser473, gene activation and stem cell proliferation

The dynamic phosphorylation of TIF1β/Ser473 during cell proliferation and differentiation (Figures 2 and 6) suggests that phosphorylation/dephosphorylation is crucial for regulating the transcriptional activity of TIF1β. During the differentiation of embryonic carcinoma cells, the intracellular distribution of TIF1β changes from diffuse nuclear staining to discrete foci and colocalizes with heterochromatin [42]. The steady-state level of TIF1β is also decreased. The reduced levels of phosphorylated TIF1β/Ser473 as well as the lower steady-state TIF1β levels in differentiated cells suggest that TIF1β may be mainly localized to heterochromatin in differentiated cells. In embryonic stem cells, TIF1β is present in complexes with various pluripotent markers, including Rex-1, Dax-1 and Nanog [22]. Since phosphorylated TIF1β/Ser473 seems to be preferentially associated with cell proliferation, it is important to determine whether it resides in these complexes. In fact, the level of phosphorylated TIF1β/Ser473 may serve as a proliferation marker. The epigenetic silencing of retrovirus transcription is caused by the binding of a TIF1β corepressor complex to retrovirus primer binding site [17]. The likely recruitment of TIF1β by a DNA-binding KRAB-box containing zinc finger protein for PBS-mediated silencing should be addressed, and the question of whether the regulation of the phosphorylation of TIF1β/Ser473 or the formation of TIF1β corepressor complex is participating in retrovirus transcription should also be investigated.

## Conclusion

Although the TIF1β co-repressor function is known to be related to HP1β, few studies have addressed the specific gene targets of TIF1β. We have found that cell cycle progression is regulated by TIF1β. The key cell cycle regulatory genes, *Cyclin A2*, *Cdc2 *and *Cdc25A *are targeted by TIF1β, and phosphorylation of TIF1β/Ser473 is associated with the activation of these genes. TIF1β/S473 phosphorylation is up-regulated during the S phase in HeLa cells and the interaction between HP1β and TIF1β is compromised when Ser473 is phosphorylated. These observations suggest that the phosphorylation of TIF1β/Ser473 may regulate gene expression by abolishing its interaction with HP1β. Thus, phosphorylation of TIF1β/Ser473 plays a crucial role in epigenetic regulation of gene expression.

## Methods

### Antibodies

Monoclonal antibody against TIF1β was generated by immunizing BALB/c mice with recombinant TIF1β corresponding to the N-terminal region (amino acids 1–250). Clone 20A1 was used throughout this investigation. Phospho-specific polyclonal antibody (S473) was produced by immunizing rabbits with KLH-conjugated peptide (VKRSRpSGEGEVC). The S473 antibody was purified by peptide-agarose affinity column chromatography. Rabbit anti-H3K9diMe and H3K4diMe antibodies were obtained from Upstate/Millipore, cyclin A and cyclin B antibodies were from BD Science, and M2 monoclonal antibody and M2 beads were from Sigma.

### Plasmids and chemicals

Mouse pCMV-FLAG-TIF1β was cloned as described by Chang et al. [43]. Site-directed mutagenesis was performed using pCMV-FLAG-TIF1β to create S473A and S473E mutants with PfuTurbo DNA Polymerase (Stratagene). The primers, based on nucleotides 1691 to 1721 of NM_005762, were designed as follows. For S473A:

Forward: 5'-GAAACGGTCCCGCGCAGGTGAGGG-3' and

Reverse: 5'-CCCTCACCTGCGCGGGACCGTTTC-3'.

For S473E:

Forward: 5'-GAAACGGTCCCGCGAAGGTGAGGG-3' and

Reverse: 5'-CCCTCACCTTCGCGGGACCGTTTC-3'.

HA-PKCδ was obtained from Dr. C. K. Chou, HA-HP1β and GST-HP1β plasmids were kindly provided by Dr. Pierre Chambon. PKC inhibitor Ro-31-8820 and calcium/calmodulin-dependent protein kinase-II (CaMK-II) inhibitor KN-93 were from BIOMOL. Casein kinase1 (CK1) inhibitor D4476 was from Calbiochem. Staurosporin, 12-O-tetradecanoylphorbol-13-acetate (TPA), thymidine, and nocodazole were from Sigma. Ser473-specific inhibitory peptide SGVKRARAGEGEVrrrrrrrrr (r stands for arginine) was obtained from Genemed Synthesis (South San Francisco, CA).

### Cell cultures

HeLa, HEK293, and HEK293T cells were cultured in Dulbecco's modified Eagle's medium plus 10% FCS and 100 units/ml penicillin/streptomycin. For synchronization at mitotic phase, HeLa cells were treated with 1 μM nocodazole for 16 hours. Cells were collected by shake-off, rinsed with PBS and cultured in complete medium. For synchronization at G1/S phase, HeLa cells were treated with 2.5 mM thymidine for 19 hours, released for 10 hours, treated for 19 hours again before release at various time points for cell cycle progression. For G1 phase synchronization, HeLa cells were serum-starved for 72 hours and then cultured in complete medium for various times. K562 cells were maintained in RPMI-1640 with 10% FCS and 100 units penicillin/streptomycin. K562 cells were induced to differentiate by treating with TPA (10 ng/ml) for 4 days [30,31].

Whole cell extracts (WCEs) were prepared by treating the cells with lysis buffer [20 mM HEPES, pH 7.4, 200 mM NaCl, 0.5% Triton-X100, 20% glycerol, 1 mM EDTA, 1 mM EGTA, 1 mM orthovanadate, and protease inhibitors (0.1 μg/ml each of aprotinin, leupeptin, pepstatin A, and 10 mM PMSF) and centrifuging at 6,000 × *g *in a microcentrifuge for 30 min at 4°C. Nuclear fractions were prepared by lysing the cells in HEPES buffer (pH 7.6) containing 10 mM NaCl, 1.5 mM MgCl_2_, 20% glycerol, 0.2 mM EDTA, 0.1% Triton X-100 and protease inhibitors for 10 min, centrifuged at 1,250 × *g *for 5 min and washed once with the same buffer. Nuclear extracts were prepared in nuclear extraction buffer containing 25 mM Tris-HCl, pH 8.0, 0.5 M NaCl, 1 mM EDTA, 10% glycerol, 0.2% NP-40 and protease inhibitors for 30 min at 4°C followed by DNase I treatment for 1 hour. For calf intestine alkaline phosphatase (CIP) treatment, 293 T cells were lysed with CIP buffer (50 mM Tris-HCl, pH 8.0, 0.1 M NaCl, 10 mM MgCl_2_, and protease inhibitors). The supernatants were incubated with 30 U of CIP at 37°C for 30 min.

### Immunoprecipitation, recombinant proteins, *in vitro *pull-down, and western blotting

WCE was pre-cleaned with protein G beads. Antibody was bound to protein G beads and mixed with pre-cleaned WCE for 2 hours at 4°C by gentle rotation. The immunocomplex was washed 3 times with immunoprecipitation buffer before SDS sample buffer was added and proteins were separated by SDS-PAGE. TIF1βs (FLAG-TIF1β/S473A, FLAG-TIF1β/S473E, and wild type FLAG-TIF1β) were co-transfected with HA-HA-HP1β into 293T cells. Nuclear extracts were used for M2 pull-down assay. GST-HP1β expressed in *Escherichia coli *DH5α was purified by binding to glutathione beads. FLAG-tagged TIF1β, TIF1β/S473A and TIF1β/S473E were expressed in 293T cells and purified by FLAG peptide elution of the M2 bead-bound proteins. For pull-down assays, glutathione bead-bound GST-HP1β was re-suspended in nuclear extraction buffer and incubated with the purified FLAG-TIF1β, FLAG-TIF1β/S473A, and FLAG-TIF1β/S473E.

### *In vitro *transcription and translation

[^35^S]Methionine-labeled FLAG-TIF1βs (FLAG-TIF1β, FLAG-TIF1β/S473A, and FLAG-TIF1β/S473E) and HA-HP1β were prepared by TnT *in vitro *transcription/translation kit (Promega).

### Chromatin immunoprecipitation assays

Chromatin immunoprecipitation (ChIP) assays were performed as described by Li et al. [44]. Briefly, synchronized G1, G1/S, early S phase, or interphase cells were treated with 1.42% of formaldehyde for 10 min at room temperature. Nuclei from 1 × 10^7 ^cells were resuspended in ChIP lysis buffer (50 mM Tris-HCl [pH 8.1], 1% SDS, 10 mM EDTA, 1× protease inhibitor cocktail) and used for each immunoprecipitation. After sonication on ice four to six times for 10 seconds followed by centrifugation for 10 min, the chromatin solution was diluted 10-fold with dilution buffer (5 μg/ml of salmon sperm DNA, 5 mg/ml BSA, 20 mM Tris-HCl [pH 8.1], 1% Triton X-100, 2 mM EDTA, 150 mM NaCl, 1× protease inhibitor cocktail). An input control of 100 μl of sonicated solution was saved and processed in parallel with the eluted immunoprecipitates beginning at the cross-link reversal step. The chromatin was pre-cleaned with protein G-agarose. Different antibodies (TIF1β monoclonal antibody, 20A1; rabbit anti-H3K9diMe and H3K4diMe antibodies) were bound to protein G-agarose first in dilution buffer containing 5 μg/ml of salmon sperm DNA and 5 mg/ml BSA. Chromatin complexes were then incubated with specific antibodies-protein G-agarose and rotated for 2–4 hours at 4°C. Immunoprecipitates were sequentially washed for 5 to 10 min in wash buffer I (20 mM Tris-HCl [pH 8.1], 2 mM EDTA, 0.1% SDS, 1% Triton X-100, 150 mM NaCl), wash buffer II (20 mM Tris-HCl [pH 8.1], 2 mM EDTA, 0.1% SDS, 1% Triton X-100, 500 mM NaCl), wash buffer III (0.25 M LiCl, 1% NP-40, 1% deoxycholate, 1 mM EDTA, 10 mM Tris-HCl [pH 8.1]), and then in TE buffer (three times). Washed beads were incubated at 65°C overnight to reverse protein-DNA cross-linking, and then each sample was treated with 10 μg of proteinase K in proteinase K buffer for 6 h at 55°C. The eluted material was combined in one tube, and the DNA was purified with the QIAquick PCR purification kit (Qiagen, Valencia, Calif.28104) and eluted in 50 μl of elution buffer. Extracted total inputs were diluted 1:50 and subjected to PCR analysis with indicated primers. Each PCR mixture contained 2 μl of immunoprecipitate or input, 1 μM each primer, 0.4 mM deoxynucleoside triphosphate mixture, 1× LA-Taq PCR buffer, and 0.25 μl LA-TaqDNA polymerase in a total volume of 50 μl. The E2F responsive primers for each promoter were as follows:

For *Cyclin A2 *promoter:

Forward: 5'-CTGCTCAGTTTCCTTTGGTTTACC-3';

Reverse: 5'-CAAAGACGCCCAGAGATGCAG-3';

and for *Cyclin E *promoter:

Forward: 5'-GCCGCCTGTCCATTCATCC-3';

Reverse: 5'-GGTCCTGTGGAGCCTGTAGCC-3'.

PCR was performed for 26 to 29 cycles with 1 min of denaturing at 94°C, annealing at 62°C, and extension at 68°C. PCR results were analyzed by agarose gel (1.5%) electrophoresis.

For real time PCR, 5 μg of FLAG-TIF1β and HA-HP-1β expression vectors were transfected into HEK 293T cells in 10-cm dish at about 20% confluence. In each precipitation, 300 μg of total cell extract was used, with 50 μg of total cell extract as input. Antibodies for FLAG (M2) or HA were used to probe recombinant TIF1β and HP1β on E2F targets of *Cdc2 *and *Cdc25A *promoters. Real-time PCR was conducted on a Roche Light Cycler 480 using the following primers for *Cdc2 *promoter:

Forward: 5'-ACAGTAGGACGACACTC-3'; and

Reverse: 5'-GGATTCACCAATCGGGTAG-3';

and the following primers for *Cdc25A *promoter:

Forward: 5'-CTAGCTGCCATTCGGT-3'; and

Reverse: 5'-CTTCGCTGTTCTCCCA-3'.

### Immunostaining

Cells on cover slips were washed with 1× phosphate-buffered saline (PBS), fixed with 2% formaldehyde for 15 min, washed with cold PBS for three times and further permeablized with 1xPBS containing 0.5% Triton-X100 for 5 min. Cells were blocked with 1% BSA for 30 min and incubated with indicated antibodies diluted in 1% bovine serum albumin/PBS, and probed with indicated primary antibodies. Alexa 594-conjugated goat anti-mouse IgG (Molecular Probes, Inc) and Alexa 488-conjugated goat anti-rabbit IgG were used as secondary immunofluorescent dyes. DAPI was used to visualize DNA. Stained cells were analyzed with a Leica TCS SP2 Confocal Spectral Microscope using a 63X/NA 1.4 oil immersion objective lens.

### Flow cytometry

Cells were collected and fixed with 70% ethanol for 30 minutes at 0°C. Cells were stained with DNA-staining solution (25 μg/ml propidium iodide, 100 μg/ml RNase A, and 0.5% Nonidet P-40 in PBS) at room temperature for 30 min. DNA content was analyzed from 10,000 cells collected with a BD Biosciences flow cytometer in conjunction with the CellQuest software. The distribution of cell populations in the cell cycle stage was gated with CellQuest analysis program (M1, subG1; M2, G1; M3, S; and M4, G2/M). The cell cycle phase distribution (in total for 100%) in each sample after CellQuest program analysis is shown. The percentage of the cell cycle phase shown in table is also presented with bar charts (by Microsoft Excel).

### RNA extraction and Real-time PCR

The level of gene expression was determined by real-time PCR. Briefly, total RNA was extracted from cells using Blue extract reagent (LTK, Inc., Taiwan) following the procedures recommended by the manufacturer. Samples of 5 μg of total RNA were reverse transcribed using M-MLV reverse transcriptase (Promega) and an oligo dT primer. The primers used for real-time PCR were for *Cyclin A2*:

Forward: 5'-GCATGTCACCGTTCCTCCTT-3';

Reverse: 5'-CAGGGCATCTTCACGCTCTAT-3';

and for *Actin*:

Forward: 5'-GAGAAAATCTGGCACCACACC-3';

Reverse: 5'-ATACCCCTCGTAGATGGGCAC-3'.

All reactions were carried out using a 7300 Real-Time PCR System (Applied Biosystems) and ABsolute™ QPCR SYBR^® ^Green Mix (ABgene, Epsom, England). The amplification was carried out as follow: initial enzyme activation at 94°C for 15 min, then 40 cycles of 94°C for 15 s and 60°C for 1 min. A total of 50 ng of each diluted reverse transcription product was used for real-time PCR in a final volume of 25 μl containing 160 nM of each specific primer and 1× ABsolute™ QPCR SYBR^® ^Green Mix (ABgene). The relative level of *Cyclin A2 *gene expression was calculated according to the comparative Ct method using the 2^-ΔΔCTCT ^formula, using the expression of *Actin *as an endogenous control.

## Authors' contributions

CWC performed most experiments and participated in the writing of the manuscript. HYC and TCH contributed to pull-down experiments with *in vitro *translated proteins. YSL performed quantitative ChIP experiments with ectopically expressed plasmids. KHH and CJC contributed to the cloning of recombinant plasmids. SCL generated 20A1 and S473 antibodies. SCL supervised and conducted the study and wrote the manuscript. All authors read and approved the final manuscript draft.
